# Understanding neurodevelopmental proteasomopathies as new rare disease entities: A review of current concepts, molecular biomarkers, and perspectives

**DOI:** 10.1016/j.gendis.2023.101130

**Published:** 2023-09-26

**Authors:** Silvestre Cuinat, Stéphane Bézieau, Wallid Deb, Sandra Mercier, Virginie Vignard, Bertrand Isidor, Sébastien Küry, Frédéric Ebstein

**Affiliations:** a Nantes Université, CHU Nantes, Service de Génétique Médicale, Nantes F-44000, France; b Nantes Université, CHU Nantes, CNRS, INSERM, l'institut du thorax, Nantes F-44000, France

**Keywords:** Biomarkers, Loss-of-function variants, Neurodevelopmental disorders, Proteasome, Rare diseases, Therapeutic targets

## Abstract

The recent advances in high throughput sequencing technology have drastically changed the practice of medical diagnosis, allowing for rapid identification of hundreds of genes causing human diseases. This unprecedented progress has made clear that most forms of intellectual disability that affect more than 3% of individuals worldwide are monogenic diseases. Strikingly, a substantial fraction of the mendelian forms of intellectual disability is associated with genes related to the ubiquitin-proteasome system, a highly conserved pathway made up of approximately 1200 genes involved in the regulation of protein homeostasis. Within this group is currently emerging a new class of neurodevelopmental disorders specifically caused by proteasome pathogenic variants which we propose to designate “neurodevelopmental proteasomopathies”. Besides cognitive impairment, these diseases are typically associated with a series of syndromic clinical manifestations, among which facial dysmorphism, motor delay, and failure to thrive are the most prominent ones. While recent efforts have been made to uncover the effects exerted by proteasome variants on cell and tissue landscapes, the molecular pathogenesis of neurodevelopmental proteasomopathies remains ill-defined. In this review, we discuss the cellular changes typically induced by genomic alterations in proteasome genes and explore their relevance as biomarkers for the diagnosis, management, and potential treatment of these new rare disease entities.

## Introduction

Intellectual disability is a neurodevelopmental disorder (NDD) occurring before adulthood which affects up to 3% of the worldwide population and is typified by both cognitive impairment and deficits in adaptive behavior.^1^, ^2^, ^3^ Approximately two-thirds of intellectual disability forms have a genetic cause with a predominance of monogenic inheritance.^4^ Thanks to the recent implementation of high-throughput sequencing technology in clinical care, the number of new genes associated with intellectual disability is growing steadily.

**Figure 1:**
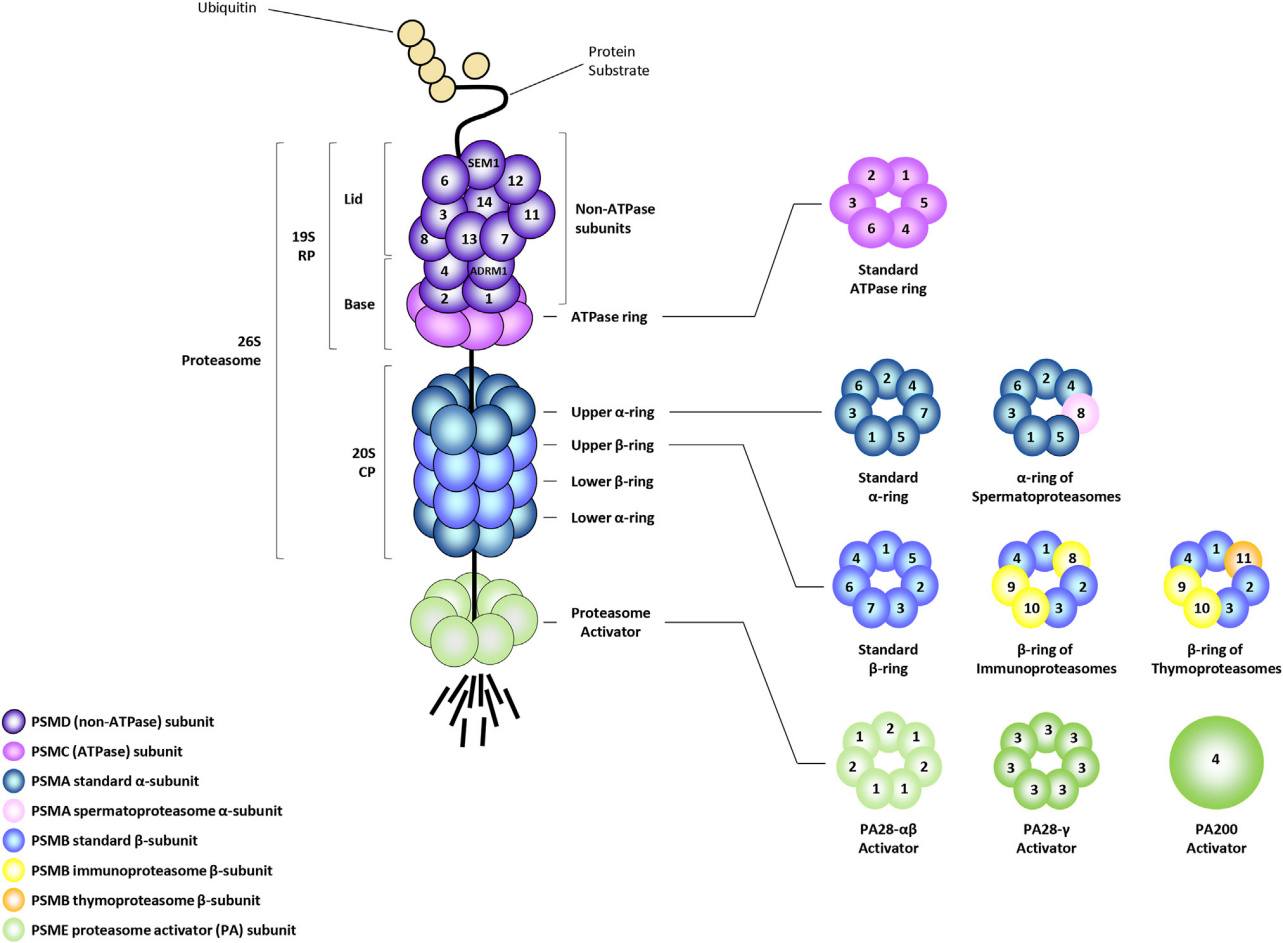
Structural organization of 26S proteasome complexes. The 26S proteasome consists of one 20S core particle (CP) associated with one end of the 19S regulatory particle (RP), which is further composed of a base and a lid, as indicated. The base of the 19S RP comprises six ATPase subunits (PSMC1 to PSMC6) and the non-ATPase subunits PSMD1, PSMD2, PSMD4, and ADRM1. The lid of the 19S RP contains the non-ATPase subunits PSMD3, PSMD6, PSMD7, PSMD8, PSMD11, PSMD12, PSMD13, PMSD14, and SEM1, as indicated. The 20S CP contains two heptameric α-rings and two heptameric β-rings, housing the standard subunits PSMA1 to PSMA7 and PSMB1 to PSMB7, respectively. In spermatoproteasomes, which are localized in the testis, PSMA7 is replaced by PSMA8. In immunoproteasomes, the catalytic standard subunits PSMB5, PSMB6, and PSMB7 are replaced by the inducible subunits PSMB8, PSMB9, and PSMB10, respectively. Thymoproteasomes in the thymus consist of immunoproteasomes in which PSMB8 is replaced by PSMB11. Eventually, proteasome activators (PA) bind to 26S proteasomes to form hybrid proteasomes. These PA include PA28-αβ, composed of PSME1 and PSME2 subunits, PA28-γ, made up of PSME3 subunits, or the PA200 protein, as indicated.

Nevertheless, in 2021, Kaplanis and colleagues suggested that about 1000 genes causing NDD have not been fully identified yet.^5^ Many — if not all — of the genes associated with NDD are involved in basic biological processes, including those regulated by the ubiquitin-proteasome system (UPS).^6^ With more than 1200 genes,^7^ the UPS is a highly conserved pathway across eukaryotic species that preserves protein homeostasis by targeting ubiquitin-tagged proteins to degradation by the proteasome.^8^ Ubiquitin is a 76-amino acid peptide that covalently modifies protein substrates destined to be removed via the sequential action of three different enzymes, namely E1 ubiquitin-activating enzymes, E2 ubiquitin-conjugating enzymes, and E3 ubiquitin ligases.^9^ Ubiquitin itself may be subjected to ubiquitination, thereby allowing the formation of polyubiquitin chains on protein substrates. It is widely accepted that proteasomes predominantly recognize polyubiquitin chains in which ubiquitin moieties are joined together via Lys48-linkages.^9^ Ubiquitin-modified proteins are typically degraded by the 26S proteasome, a giant protein complex composed of a 20S core particle (CP) capped at one end by a 19S regulatory particle (RP)^10^ (Fig. 1). The 19S RP can be further divided into two parts, namely the lid and the base.^10^ As depicted in Fig. 1, the lid is made up of eight scaffolding subunits — *PSMD3*/Rpn3, *PSMD12*/Rpn5, *PSMD11*/Rpn6, *PSMD6*/Rpn7, *PSMD7*/Rpn8, *PSMD13*/Rpn9, *PSMD8*/Rpn12, and *SEM1*/Rpn15 — and one deubiquitinating enzyme *PSMD14*/Rpn11.^10^ As for the base of the 19S RP, it is formed by the association of six different AAA+ ATPase subunits (Rpt1-6 encoded by *PSMC1-6* genes) with the four non-ATPase subunits *PSMD2*/Rpn1, *PSMD1*/Rpn2, *PSMD4*/Rpn10, and *ADRM1*/Rpn13^10^ (Fig. 1). In contrast to the lid whose subunits may self-assemble, the biogenesis of the base relies on four assembly chaperones, namely *PSMD9*/p27, *PSMD10*/p28, *PSMD5*/S5b, and *PAAF1*/Rpn14.^11^ The base and the lid then associate following their individual maturation.^11^ One major function of the 19S RP consists of sensing ubiquitin-modified proteins via the *PSMD4*/Rpn10 and *ADRM1*/Rpn13 subunits which serve as ubiquitin receptors. Following recognition and binding, protein substrates are then deubiquitinated by *PSMD14*/Rpn11, USP14, and UCHL5 and finally unfolded by the six AAA+ ATPase *PSMC1-6*/Rpt1-6 before translocation into the 20S CP.^12^

As illustrated in Figure 1, the 20S CP is built by the juxtaposition of four heptameric rings consisting of two outer α-rings and two inner β-rings each composed of seven different α- and β-subunits encoded by the *PSMA1-7* and *PSMB1-7* genes, respectively.^10^ The 20S CP arises from the dimerization of 16S α/β heterodimer precursor complexes which themselves assemble with the assistance of the four chaperones PAC1-4 encoded by the *PSMG1-4* genes and the proteasome maturation protein POMP encoded by *POMP*.^11^ The 20S CP ensures the degradation of translocated protein substrates into short peptides via the catalytic β-subunits (*PSMB6*/β1, *PSMB7*/β2, *PSMB5*/β5 carrying caspase-like, trypsin-like, and chymotrypsin-like activities, respectively).^13^ Depending on the composition of the β-ring and/or association with other RP than 19S, several proteasome isoforms may arise. These include notably immunoproteasomes in which the *PSMB6*/β1, *PSMB7*/β2, and *PSMB5*/β5 standard subunits have been replaced with the *PSMB9*/β1i, *PSMB10*/β2i, and *PSMB8*/β5i inducible ones^14^ (Fig. 1). While immunoproteasomes are constitutively expressed in immune cells, their biogenesis can be induced in non-immune cells following exposure to inflammatory stimuli such as interferons (IFN).^15^ It is understood that immunoproteasomes degrade protein substrates faster than their standard counterparts, a property that makes them critical regulators of protein homeostasis and MHC class I antigen presentation.^16^^,^^17^ As depicted in Figure 1, the 20S CP of proteasomes may contain other atypical subunits, namely *PSMA8*/α4s and *PSMB11*/β5t, which are exclusively found in the testis and thymus, respectively.^18^^,^^19^ *PSMA8*/α4s is a crucial component of the spermatoproteasome, responsible for the breakdown of meiotic proteins like RAD51 and RAP1, a process essential for the progression of meiosis during spermatogenesis.^20^ On the other hand, *PSMB11*/β5t is a part of the thymoproteasome, and its role involves providing MHC class I-restricted peptides, which facilitates the positive selection of CD8^+^ T cells,^21^ even though the precise mechanisms underlying this process remain poorly understood.

Independently of the α- and β-subunits, several additional particles other than the 19S RP may bind to the 20S CP to form extra proteasome types. For instance, as illustrated in Figure 1, most 20S complexes in the cell are associated with a PA28-αβ ring made up of three *PSME1*/PA28-α- and four *PSME2*/PA28-β subunits^22^ or four *PSME1*/PA28-α- and three *PSME2*/PA28-β subunits^23^ to build PA28-capped proteasomes. It has been shown that PA28-20S complexes are capable of degrading protein substrates such as oxidized proteins in an ATP- and ubiquitin-independent fashion.^24^ Apart from PA28-αβ, proteasomes may also associate with *PSME3*/PA28γ or *PSME4*/PA200 (Fig. 1) to modulate the turnover of nuclear proteins, in particular cell cycle regulators and histones, respectively.^25^^,^^26^ Less prominent regulators include ECM29 and PI31 whose binding to proteasomes may exert various functional consequences such as 26S complex disassembly, redistribution, and inhibition.^27^^,^^28^

## Genetics of proteasomopathies

The term “proteasomopathies” is a recent portmanteau used to design disorders caused by proteasome dysfunction.^29^ Proteasomopathies thus represent a rather large group of heterogenous diseases which, depending on their molecular etiology, may be further classified into different subtypes. In this review, we will focus on primary proteasomopathies, namely on congenital defects resulting from *de novo* and/or inherited variants affecting at least one of the 52 genes encoding proteasome subunits, assembly factors, and/or regulators. These diseases are rare or even ultra-rare with so far less than 120 cases reported worldwide. This rarity is in sharp contrast to secondary proteasomopathies, which include well-known late-onset idiopathic proteasome deficiencies such as neurodegenerative diseases whose genetic component is much less well-defined.

As mentioned above, primary proteasomopathies are early-onset disorders caused by genomic alterations in proteasome genes. Intriguingly, proteasome lesions convey two distinct clinical phenotypes characterized by either systemic autoinflammation or neurodevelopmental delay. Although these manifestations are not strictly mutually exclusive, they are considered to underlie two distinct disease entities which we will refer to as (i) autoinflammatory and (ii) neurodevelopmental proteasomopathies. To date, the molecular basis for the dual phenotype is not understood. A peculiarity that is even harder to follow given that these two phenotypes cannot be mapped to specific proteasome variants and/or genes.

## Autoinflammatory proteasomopathies (CANDLE/PRAAS)

**Figure 2:**
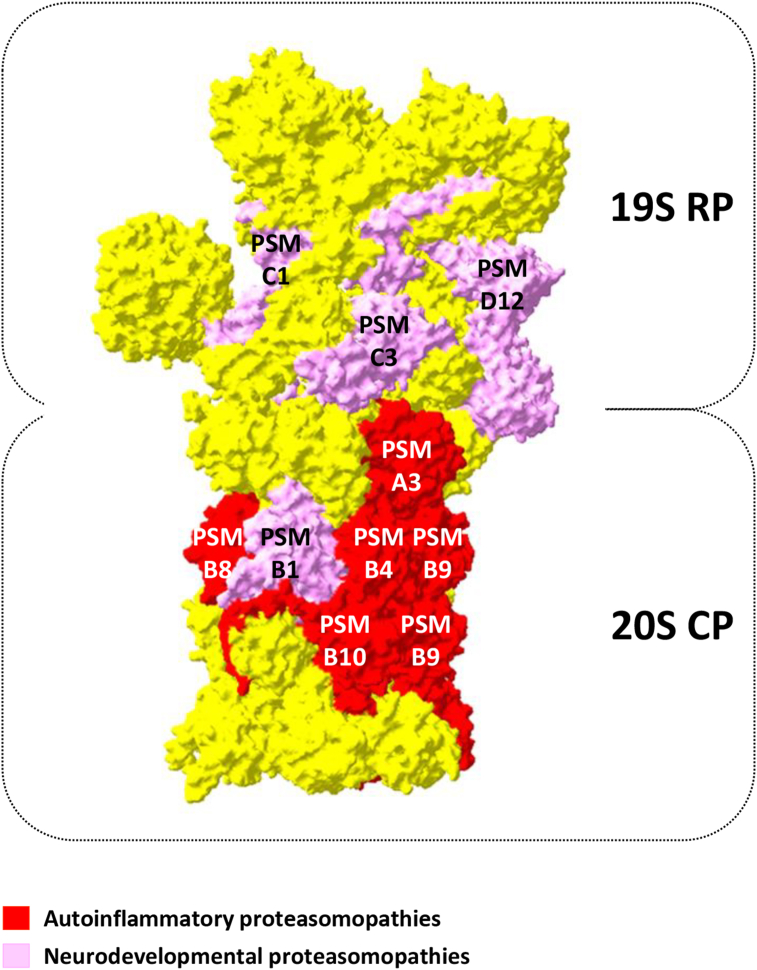
Localization of the proteasome subunits subjected to genetic lesions in proteasomopathies. Representation of the mutant proteasome subunits found in CANDLE/PRAAS (red) and neurodevelopmental proteasomopathies (pink) within the 26S proteasome complex using the structure of Zhu et al (PDB ID: 7QY7, to be published). For the sake of clarity, the standard subunits of the original structure, namely PSMB5, PSMB6, and PSMB7, have been replaced with their respective inducible counterparts: PSMB8, PSMB9, and PSMB10 to depict immunoproteasomes.

From a chronological point of view, autoinflammation was the first clinical manifestation associated with proteasome loss-of-function. In 2010, Agarwal et al reported the first p.(Thr75Met) homozygous missense variant in the *PSMB8* proteasome gene^30^ in patients suffering from an autoinflammatory syndrome named joint contractures, muscle atrophy, microcytic anemia, and panniculitis-induced lipodystrophy syndrome.^31^ To the best of our knowledge, approximately 36 variants have been identified in seven proteasome genes in 38 individuals, all presenting with typical signs of systemic autoinflammation (Table 1). Meanwhile, two different names have been given to these conditions, namely chronic atypical neutrophilic dermatosis with lipodystrophy and elevated temperature (CANDLE) and proteasome-associated autoinflammatory syndromes (PRAAS).^32^ Strikingly, genes frequently altered in CANDLE/PRAAS include the three genes coding for the inducible subunits *PSMB8*/β5i, *PSMB9*/β1i, and *PSMB10*/β2i, suggesting that these diseases were specifically caused by immunoproteasome loss of function. It became, however, rapidly evident that CANDLE/PRAAS were not exclusively associated with immunoproteasome genes and that lesions in other α- and/or β-subunits or even proteasome assembly factors (POMP, PSMG2) could trigger the disease as well (Table 1). Remarkably, as shown in Figure 2, all proteasome subunits affected in CANDLE/PRAAS are localized within the 20S CP, suggesting that the disease selectively preserves the 19S RP. Except for four cases caused by *de novo* heterozygous variants in *PSMB9* or *POMP*, the concomitant presence of two variants is required to cause the inflammatory phenotype, following a recessive monogenic — homozygous or compound heterozygous — or double heterozygosity inheritance (Table 1). This observation suggests that, apart from the four *PSMB9* and *POMP* outliers, which likely function as dominant-negative variants, most proteasome lesions can be easily rescued by the second wild-type allele, indicating that the majority of CANDLE/PRAAS are recessive disorders. Nevertheless, as discussed above, the current lack of consistency across variants makes it too premature to propose a model of genetic architecture for these diseases.

**Table 1:** Loss-of-function variants in associated proteasome genes found in autoinflammatory (CANDLE/PRAAS) and neurodevelopmental proteasomopathies.

		Gene	Function	Gene variant	Protein variant	Cases	Inheritance	Reference
Autoinflammatory proteasomopathies	Monogenic	*PSMB4*	β-subunit (non-catalytic)	c.–9G>A c.634_642del	5′ UTR region p.(D212_V214del)	1	Compound heterozygosity	32
				c.231del c.494+17A>G	p.(L78Wfs∗31) p.(?)	1		50
		*PSMB8*	β-subunit (catalytic)	c.590G>T	p.G197V	3	Homozygosity	51
				c.602G>T	p.G201V	5		52
				c.405C>A	p.C135∗	1		146
				c.224C>T	p.T75M	4		30
				c.349A>G	p.M117V	1		147
				c.271G>A	p.A92T	1		148
				Undisclosed	Undisclosed	1		149
				c.224C>T c.271G>A	p.T75M p.A92T	1	Compound heterozygosity	150
				c.373C>T c.355G>A	p.R125C p.D119N	1		151
				c.163C>T c.352T>C	p.Q55∗ p.S118P	1		152
				c.275C>T c.313A>C	p.A92V p.K105Q	1		153
				c602G>T c.389delT	p.Gly201Val p.129Argfs∗27	1		154
		*PSMB9*	β-subunit (catalytic)	c.467G>A	p.G156D	3	Heterozygosity	105 53
		*PSMB10*	β-subunit (catalytic)	c.41T>C	p.F14S	1	Homozygosity	54
		*POMP*	Proteasome assembly factor	c.344_345insTTTGA	p.E115Dfs∗20	1	Heterozygosity	
				c.342_348delinsACC	p.F114Lfs∗18	1		55
				c.334_335delAT	p.I112Wfs∗3	1		
				c.326dupA	p.D109Efs∗2	1		155
		*PSMG2*	Proteasome assembly factor	c.666_667delGT c.675T>G	p.Y223Sfs∗2 p.N225K	1	Compound heterozygosity	56
	Double heterozygosity	*PSMB4* *PSMB8*	β-subunit (non-catalytic) β-subunit (catalytic)	c.666C>A c.313A>C	p.Y222∗p.K105Q	2	Double heterozygosity	32
		*PSMB8* *PSMA3*	β-subunit β-subunit	c.224C>T c.404+2T>C c.224C>T c.696_698del	p.T75M p.H111Ffs∗10 p.T7E5M p.R233del	1 1		
		*PSMB9* *PSMB4*	β-subunit β-subunit	c.494G>A c.44insG	p.G165D p.P16Sfs∗45	2		
Neurodevelopmental proteasomopathies	Monogenic	*PSMB1*	β-subunit	c.307T>C	p.Y103H	2	Homozygosity	156
		*PSMC1*	ATPase subunit	c.983T>C	p.I328T	1	Homozygosity	39
				Deletion	Expected haploinsufficiency	3	Heterozygosity	40
		*PSMC3*	ATPase subunit	c.1127 + 337A>G	p.S376Rfs15∗	3	Homozygosity	37
				c.511C>T	R171W	1	Heterozygosity	38
				c.523A>G	M175V	1		
				c.686C>T	P229L	1		
				c.710C>T	A237V	1		
				c.775A>G	M259V	1		
				c.776T>C	M259T	1		
				c.782T>C	I261T	6		
				c.784G>A	G262R	1		
				c.806G>C	R269P	1		
				c.859G>C	E287Q	1		
				c.910C>T	R304W	4		
				c.910C>G	R304G	1		
				c.915G>T	E305D	1		
				c.929T>C	M310T	1		
				c.1147G>A	E383K	1		
		*PSMD12*	non-ATPase subunit	c.367C>T	p.R123∗	1	Heterozygosity	35
						8		33
				c.1274T>G	p.L425∗	1		33
				c.601C>T	p.R201∗	2		33 35
				c.909−2A>G	p.(?)	1		33
				Deletion	Expected haploinsufficiency	10		33 34
				c.544C>T	p.R182∗	1		34
				c.1071_1072delAG	p.R357fs∗3	1		
				c.435_438del	p.T146Kfs∗3	1		
				c.937G>T	p.E313∗	1		
				c.508_509del	p.Q170Gfs∗40	2		
				A>T	p.L149∗	1		
				c.316C>T	p.Q106∗	1		
				c.1033G>T	p.E345∗	1		
				c.1083+1G>A	p.(?)	1		
				c.1246C>T	p.Q416∗	1		
				c.526del	p.S176Qfs∗15	1		
				c.1162−1G>A	p.(?)	1		
				c.1300del	p.S434Hfs∗2	1		
				c.795+1G>A	p.(?)	1		
				c.148_149del	p.L50Gfs∗26	1		
				c.435_438del	p.T146Kfs∗3	1		
				c.544C>T	p.R182∗	1		
				c.1060_1061del	p.L354Efs∗ 6	1		
				c.906C>A	p.Y302∗	1		
				c.865C>T	p.R289∗	2		57

## Neurodevelopmental proteasomopathies

In 2017, a new form of NDD caused by loss-of-function heterozygous variants in the *PSMD12* proteasome gene was reported.^33^ This came as a surprise since it was initially assumed that autoinflammation was the only phenotype conferred by proteasome variants. In this dominant neurodevelopmental proteasomopathy, originally referred to as Stankiewicz–Isidor syndrome, mild to severe intellectual disability with radial ray, cardiac and renal malformations represented, among other possible manifestations, an inconstant but evocative association.^34^^,^^35^

Over the last five years, the concept of neurodevelopmental proteasomopathies has been strengthened by the identification of new variants in further proteasome genes in patients presenting with similar neurological manifestations. To date, 46 proteasome lesions in 78 individuals have been uncovered (Table 1). Up to this point, a total of six neurodevelopmental proteasomopathies have been described, involving the four subunits *PSMB1*/β6, *PSMC1*/Rpt2, *PSMC3*/Rpt5, and *PSMD12*/Rpn5 (Table 2). In contrast to their autoinflammatory counterparts, neurodevelopmental proteasomopathies are mostly monogenic dominant disorders arising from *de novo* mutations in proteasome genes. Indeed, approximately 85% of individuals suffering from these diseases carry heterozygous variants within the *PSMD12* or *PSMC3* genes (Table 2). Recessive forms, reported in 8/78 patients with *PSMB1*, *PSMC1*, or *PSMC3* biallelic variants, represent only 10% of neurodevelopmental proteasomopathies.

**Table 2:** Clinical features of neurodevelopmental proteasomopathies.

	Stankiewicz–Isidor syndrome with type I IFN production	Disease with ID, microcephaly, and developmental delay	Neurosensory syndrome combining deafness and cataract	Neurodevelopmental disorder with type I IFN production	Neurological syndrome	Developmental and language delay	Total
*Gene involved*	*PSMD12*	*PSMB1*	*PSMC3*		*PSMC1*		
Inheritance	(33/45) D*e novo*; (9/45) dominant; (3/45) n.a	(2/2) Recessive	(3/3) Recessive	(21/22) *De novo* (1/22) n.a	(3/3) Recessive	(1/3) D*e novo*; (1/3) dominant; (1/3) n.a	
References	33, 34, 35, 57	156	37	38	39	40	
Number of published patients	45	2	3	22	3	3	78
Gender (Age at last assessment, years)	24 M/21 F (1–42)	2 F (22–35)	3 M (7–16)	13 M/9 F (age n.a)	3 M (3–20)	3 M (3–6)	45 M/78 (57.6%)
Variants	Heterozygous truncating variant	Homozygous missense	Homozygous splice variant	Heterozygous missense	Homozygous missense	Heterozygous deletions	
*Neurodevelopment*							
Developmental delay	100% (45/45)	100% (2/2)	100% (3/3)	100% (21/21)	100% (3/3)	100% (3/3)	77/77 (100%)
Speech delay	94% (32/34)	100% (2/2)	100% (3/3)	100% (18/18)	100% (3/3)	100% (3/3)	61/63 (97%)
Motor delay	50% (17/34)	100% (2/2)	n.r	78% (14/18)	100% (3/3)	0% (0/3)	36/60 (60%)
Intellectual disability; severity	85% (34/40); mild to severe	100% (2/2); severe	100% (3/3); mild to severe	88% (15/17); n.a	100% (3/3); severe	33% (1/3); mild	58/68 (85%)
Abnormal behaviors	67% (25/37)	100% (2/2)	66% (2/3)	53% (9/17)	n.a	100% (3/3)	41/62 (66%)
Autistic features	31% (13/42)	0% (0/2)	66% (2/3)	n.a	n.a	33% (1/3)	16/50 (32%)
ADHD features	12% (5/42)	50% (1/2)	0% (0/3)	n.a	n.a	33% (1/3)	7/50 (14%)
*Neurological features*							
Epilepsy	13% (6/45)	0% (0/2)	0% (0/3)	25% (5/20)	0% (0/3)	0% (0/3)	11/76 (14%)
Regression	n.r	n.r	n.r	n.r	n.r	n.r	
Other neurological findings	n.r	Hypotonia 100% (2/2)	Ataxia 100% (3/3); peripheral polyneuropathy of lower limbs 66% (2/3)	n.r	Spastic tetraplegia 100% (3/3); chorea 100% (3/3); central hypotonia 100% (3/3)	n.r	
Abnormal brain MRI	21% (4/19); pineal cyst, cerebral atrophy, hypomyelination, periventricular nodular heterotopia	n.a	0% (0/3)	79% (11/14); additional description n.a	100% (2/2); ventriulomegaly	0% (0/1)	
*Morphological features*							
Intrauterine growth restriction	37% (9/24)	n.r	n.r	n.r	n.r	n.r	
Other antenatal features	n.r	n.r	n.r	n.r	n.r	n.r	
Growth failure	40% (4/10)	100% (2/2)	n.r	50% (9/18)	n.r	n.r	15/30 (50%)
Abnormal facial shape	97% (44/45)	100% (2/2)	100% (3/3)	89% (17/19)	100% (3/3)	66% (2/3)	71/75 (95%)
Microcephaly	21% (9/42)	100% (2/2)	n.r	38% (6/16)	100% (3/3)	n.r	20/63 (32%)
Macrocephaly	7% (3/42)	0% (0/2)	n.r	13% (2/16)	0% (0/3)	n.r	5/63 (8%)
Skeletal abnormalities	61% (21/34); including thumb anomalies: 29% (10/34)	n.r	n.r	71% (10/14)	n.r	n.r	
*Sensorial features*							
Hearing impairment	18% (6/34)	100% (2/2)	100% (3/3)	8/18 (44%)	100% (3/3)	n.r	22/60 (37%)
Ophthalmological abnormalities	74% (20/27); including strabismus, coloboma, Peters anomaly, corneal opacity	n.r	Early onset cataract: 100% (3/3); strabismus: 66% (2/3)	n.a	n.r	n.r	
*Visceral features*							
Cardiac	44% (15/34)	n.r	n.r	59% (10/17)	33% (1/3)	n.r	26/54 (48%)
Renal	50% (15/30)	n.r	n.r	29% (4/14)	33% (1/3)	n.r	20/47 (42%)
Genital	40% (12/30)	n.r	n.r	n.r	100% (3/3)	n.r	15/33 (45%)
Other findings	Congenital pancytopenia: 2% (1/45); cleft palate: 2% (1/45)	n.r	Semicircular canal malformations 33% (1/3); depigmented hairs of lower limbs 66% (2/3)	Tumors: 11% (2/18) craniopharyngioma and neuroblastoma; orofacial clefts: 10% (2/19)	n.r	Sleep irregularities: 66% (2/3)	
*Inflammatory symptoms*							
Chilblains	5% (2/36)	n.r	n.r	n.r	n.r	n.r	
Urticarial skin rashes	3% (1/36)	n.r	n.r	n.r	n.r	n.r	
Congenital uveitis	3% (1/36)	n.r	n.r	n.r	n.r	n.r	
Subcutaneous calcifications	n.r	n.r	100% (3/3)	n.r	n.r	n.r	

Notes: n.r: not reported; n.a: not assessed.

As shown in Table 2, the hallmark of these six disorders is mild to severe developmental delay (100%), characterized by predominant speech delay (97%) and intellectual disability (85%), very often accompanied by behavioral abnormalities (66%), mainly autism spectrum disorder, and motor delay. Furthermore, nearly all patients (95%) display dysmorphic facial features (Table 2), although these attributes are highly diverse and do not follow clinically recognizable patterns. Besides these manifestations, the clinical spectrum of these disorders is remarkably wide, and the phenotype associated with these variants varies significantly among individuals. Notably, a significant proportion of patients exhibit multi-system malformations, likely indicating the ubiquitous expression of the proteasome variants and their critical impact on human development. As shown in Table 2, these visceral malformations can affect the brain, heart, and kidneys, as well as the reproductive and skeletal systems. Notably, visceral malformations have not been reported in forms with *PSMB1* or *PSMC3* biallelic variants nor with *PSMC1* deletions (Table 2). However, these observations should be interpreted with caution, given the limited number of cases reported for these forms.

It seems that at least two of the six disorders can be considered as recognizable syndromes. One of these is Stankiewicz–Isidor syndrome, which is consistently characterized by intellectual disability, speech delay, several abnormalities, including the inconstant but evocative association of thumb anomalies with sensorineural hearing loss as well as signs of inflammation, such as chilblains, urticarial skin rash, and congenital uveitis (Table 2). The second is the recessive disorder associated with *PSMC3* variants, which can be uniformly defined by developmental delay, speech delay, early onset cataract, and sensorineural hearing loss (Table 2). Both syndromes highlight the relatively high frequency of hearing impairment observed in neurodevelopmental proteasomopathies, in line with the role of the proteasome in inner ear development.^36^

A particularly intriguing observation is that mutations within the same *PSMC3* gene can give rise to two distinct clinical phenotypes. While one results in a recessive neurosensory syndrome characterized by intellectual disability, ataxia, peripheral polyneuropathy, deafness, and cataract, attributed to a deep intronic mutation,^37^ the other one presents a dominant form of NDD featuring type I IFN production, caused by missense variants.^38^ Genomic alterations impacting the *PSMC1* gene exhibit a similar phenomenon with recessive missense variants causing a neurological syndrome typified by severe intellectual disability, chorea, and spastic tetraplegia,^39^ and heterozygous deletions leading to a milder form of NDD without motor delay.^40^ Furthermore, while both dominant and recessive syndromes lead to impaired neurodevelopment, it is noteworthy that the recessive variants seem to affect the central nervous system (CNS) in a broader manner, not restricted to cognitive functions (Table 2). However, it is important to acknowledge that the interpretation of these findings is constrained by the limited number of patients. As such, further investigations with larger patient cohorts are warranted to validate and strengthen these observations.

Another compelling observation pertains to two patients harboring *PSMC3* heterozygous missense variants, both of whom have been documented to develop tumors,^38^ specifically craniopharyngioma and neuroblastoma. This raises an important question regarding a potential predisposition to cancer in individuals affected by these diseases. Given the possibility of cancer susceptibility in these cases, it is worth mentioning that chronic inflammation, particularly type I IFN, is a well-known factor contributing to genomic instability and carcinogenesis.^41^ It is plausible that sustained inflammation may play a role in explaining the occurrence of tumors in some patients with *PSMC3* missense variants.

As shown in Figure 2, the observation that only one (*i.e.*, p.Tyr103His in *PSMB1*/β6) of the forty-three lesions identified in individuals with neurodevelopmental proteasomopathies affect the 20S CP strongly suggests that these disorders are essentially diseases of the 19S RP, an assumption which remains to be confirmed in future studies. One should also emphasize that the number of genes associated with neurodevelopmental proteasomopathies highlighted in this review is likely underestimated. Indeed, exome sequencing of large NDD cohorts recently suggested the implication of several additional strong candidates including *PSMA7*/α4, *PSMD10*/p28, or *PSMC5*/Rpt6 whose *de novo* missense variants were found to be statistically enriched in patients.^5^^,^^42^^,^^43^ Likewise, haploinsufficiency of certain proteasome genes was suggested to contribute to the cognitive phenotype of several microdeletion syndromes, such as *PSMD10*/p28 in Xq22.3, *PSMD11*/Rpn6 in 17q11.2, or *PSMD14*/Rpn11 in 2q24.2 deletions, respectively.^44^, ^45^, ^46^, ^47^, ^48^ While these studies did not conclusively establish the pathogenicity of these alterations in functional studies, the observation that most of these genes encode ATPases and non-ATPase subunits support the 19S RP hypothesis of these diseases, which is further reinforced by the ongoing constitution of NDD patient cohorts with variants in the *PSMC5* and *PSMD11* genes (manuscripts in preparation). Though this specificity seems difficult to pinpoint, recent research by Sun et al^49^ has provided valuable insights into the propensity of 19S subunits to initiate neurodevelopmental proteasomopathies. This study indeed proposes that synapses have a significant enrichment of “free” 19S RP that perform functions independent of the proteasome. Consequently, it is plausible that neurons are more vulnerable to changes in the 19S subunits compared with other cell types, as discussed later.

As noted earlier, one major hurdle to our understanding of the molecular pathogenesis of neurodevelopmental proteasomopathies remains their clinical heterogeneity, with a phenotype spectrum ranging from mild neurodevelopmental impairment to severe neurosensory deficits. Furthermore, the associated malformations seem variable and inconsistent, making it difficult for now to identify a recognizable clinical pattern. Very few inherited variants reported for these diseases reveal considerable intrafamilial variability in the severity of the neurocognitive impairment they trigger.^35^ For these reasons, and given the very limited number of cases described so far, a genotype–phenotype correlation of these diseases remains difficult to establish.

## Biomarkers for neurodevelopmental proteasomopathies

**Figure 3:**
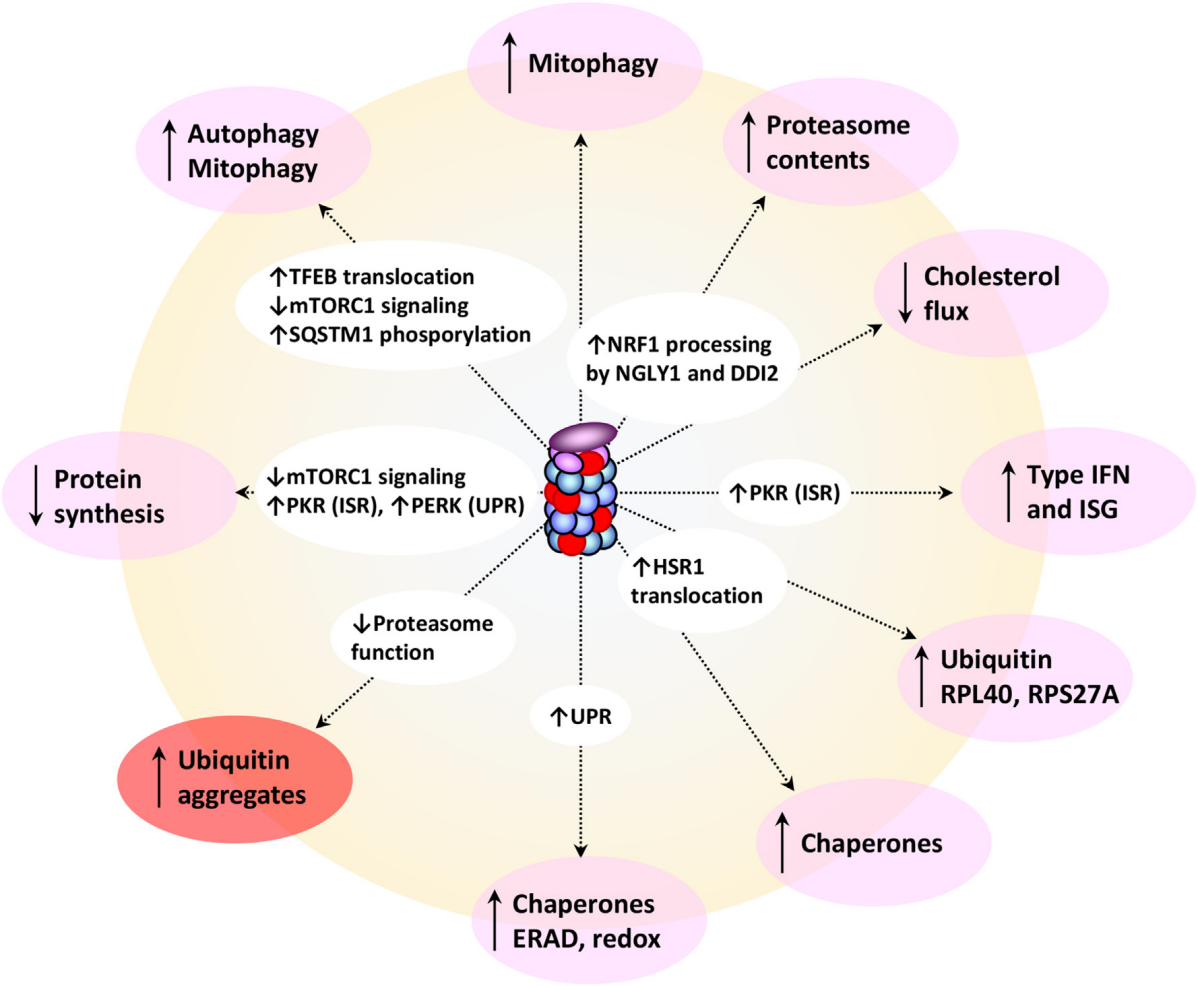
Proteasome dysfunction results in several cellular consequences which can be used as biomarkers for screening proteasomopathies. Proteasome carrying loss-of-function mutant subunits (red) exhibit decreased activity which ultimately results in increased protein aggregation (light red), as indicated. Compromised proteasome function is typically accompanied by parallel engagement of adaptation programs (pink) including a rise of autophagy, mitophagy, proteasome contents, type I IFN, cholesterol esterification, ubiquitin, and molecular chaperone synthesis as well as reduction of protein biosynthesis. Both protein aggregation and compensatory pathways can be used as indicators for proteasomopathies.

A common feature of all pathogenic proteasome gene variants is their propensity to generate proteotoxic stress, because of proteasome loss of function. As discussed below, this is best exemplified by the accumulation of ubiquitin-modified protein aggregates. It should, however, be emphasized that virtually all tissues are equipped with control quality systems capable of sensing and correcting protein homeostasis perturbations. Accordingly, this implies that overactivation of either one of these processes may serve as potential biomarkers for neurodevelopmental proteasomopathies as well (Fig. 3).

## Protein aggregation

Ubiquitin-positive inclusions have been consistently documented in various cell types across individuals carrying proteasome variants, regardless of their clinical phenotype.^32^, ^33^, ^34^^,^^37^^,^^38^^,^^50^, ^51^, ^52^, ^53^, ^54^, ^55^, ^56^, ^57^ These ubiquitin-modified aggregates are known to originate primarily from defective ribosomal products, namely newly synthesized proteins that have failed to reach their native and functional three-dimensional conformation.^58^ It is estimated that 20%–30% of the translation products are defective ribosomal products destined for degradation,^59^ thereby implying that tissues with a high demand for protein synthesis are more sensitive to proteasome dysfunction and proteotoxic stress. Notably, recent studies have shown that the brain, liver, and pancreas are among the organs with the highest rates of protein synthesis in the human body.^60^^,^^61^ As such, uncontrolled accumulation of ubiquitin-protein conjugates in the CNS may represent one of the most reliable biomarkers of these rare diseases.

## Increased autophagy

Not surprisingly, dysfunction of the UPS has been shown to stimulate the second main degradative pathway in eukaryotic cells, the autophagy-lysosomal pathway.^62^ Autophagy is a highly conserved intracellular machinery that sequesters cytosolic substrates within autophagosomes prior to their degradation in lysosomes.^63^^,^^64^ Depending on the presence of recognition elements carried by substrates, autophagy may be selective or non-selective. For instance, selectivity may be achieved via the modification of cargos with Lys63-linked polyubiquitin chains which are then recognized by autophagy receptors such as SQSTM1/p62.^65^ The latter, in turn, delivers ubiquitinated substrates to nascent autophagosomes thanks to its ability to interact with LC3 precursors and/or processed forms.^66^ The crosstalk between UPS and autophagy is largely regarded as a compensatory mechanism enabling the cell to cope with accumulating ubiquitin-protein conjugates when either one of these systems becomes defective. The mechanisms by which UPS impairment stimulates autophagy are diverse and rely on both gene expression and post-translational modifications. For instance, proteasome inhibition induces the expression of key components of the autophagy pathway via the transcription factor TFEB.^67^, ^68^, ^69^, ^70^ In addition, proteasome impairment activates the autophagy-lysosomal pathway by promoting the phosphorylation of SQSTM1/p62 at Ser403 by various kinases, including PINK1, p38δ, or TBK1,^71^, ^72^, ^73^ a process that increases the binding affinity of SQSTM1/p62 for its ubiquitin-modified cargos.^74^ A third mechanism involves mTOR down-regulation, which occurs following proteasome inhibition due to a decreased supply of peptides and amino acids.^75^ Reduced mTOR signaling then stimulates autophagy by shifting the phosphorylated forms of the inhibitory kinase ULK1 to the unphosphorylated ones.^76^

Importantly, the substrate spectrum of autophagy is by far larger than that of the UPS, as it includes intracellular organelles, such as ribosomes and mitochondria.^77^ Accordingly, elevated degradation rates of mitochondria by autophagy, a process referred to as “mitophagy”, have been frequently observed in cells treated with proteasome inhibitors.^78^ Supporting this notion, we could also show that individuals with Stankiewicz–Isidor syndrome exhibited a higher mitochondrial turnover than healthy individuals.^34^ Given the importance of mitochondrial respiration in neuronal function,^79^ it is highly likely that persistent mitophagy caused by proteasome loss-of-function variants might be a key driver in the pathogenesis of neurodevelopmental proteasomopathies.

## NRF1 activation

Besides alerting autophagy, defective proteasomes can augment their own synthesis, a process that relies on the endoplasmic reticulum (ER) membrane-resident protein NRF1 (also referred to as TCF11). NRF1 is a highly glycosylated short-lived transcription factor with a half-life of approximately 12 min under normal conditions.^80^ Upon proteasome impairment, NRF1 becomes stabilized and undergoes proteolytic cleavage by the aspartyl protease DDI2, whereby the C-terminal part translocates into the nucleus and induces the transcription of proteasome genes.^80^ In this regard, we were able to demonstrate that the NRF1-DDI2 axis is persistently activated in patients carrying *PSMC3* variants,^37^ a process creating a vicious circle in which defective proteasomes up-regulate themselves. Interestingly, target genes of NRF1 also include genes related to autophagy (*i.e.*, SQSTM1/p62) and mitophagy.^81^ In addition, in the nucleus, NRF1 represses the expression of *SOAT1*, a gene coding for acyl-coenzyme A: cholesterol acyltransferase 1, an enzyme that catalyzes cholesterol esterification.^82^ This observation has led to the concept that NRF1 may act as a cholesterol sensor, being retained at the ER under conditions of high membrane cholesterol concentrations, thereby reducing the levels of free cholesterol. In addition, Bartelt and colleagues have recently shown that cold temperature activates NRF1 to induce a transcriptional program promoting brown adipose tissue formation.^83^

Altogether, these studies point to an unexpected cross-regulation of proteasome function and lipid metabolism associating proteasome loss of function with increased mobilization of free cholesterol. This notion is in line with the fact that patients with CANDLE/PRAAS suffer from lipodystrophy.^32^ Intriguingly, individuals suffering from neurodevelopmental proteasomopathies seem clinically devoid of lipid metabolism alterations. Their skeletal malformations, however, may be related to impaired brown adipose tissue function, whose proper functioning has been positively correlated with bone anabolism.^84^^,^^85^

## Activation of the heat-shock response

Early studies have revealed that cells treated with proteasome inhibitors rapidly mount a heat-shock response.^86^ Heat-shock response is traditionally viewed as a highly conserved process destined to induce the transcription of genes coding for stress proteins and heat-shock proteins in order to preserve protein homeostasis under challenging conditions. Prominent heat shock proteins induced by the heat-shock response include HSP70 and HSP90, whose function consists of protecting and/or refolding proteins using energy provided by ATP hydrolysis. Activation of the heat-shock response relies on the transcription factor HSF1 which, under normal conditions, is retained in the cytosol. As of now, the exact molecular mechanisms responsible for HSF1 translocation into the nucleus in response to proteasome inhibition remain unknown. Interestingly experiments in yeast have revealed that, besides molecular chaperones, target genes of HSF1 include components of the UPS^87^^,^^88^ among which the ubiquitin E3 ligases Hul5, Rsp5, and UBE2O.^89^, ^90^, ^91^ Of note, the mammalian ortholog of yeast Hul5, UBE3C, restores ubiquitin chains on partially proteolyzed substrates at proteasomes, a process understood to augment the processivity of proteasomes.^92^^,^^93^ Similarly, the mammalian ortholog Rsp5, NEDD4 supports protein breakdown by shuttling ubiquitin-modified protein to proteasomes for their subsequent degradation. Finally, HSF1-induced genes include the ubiquitin fusion genes *UBB*, *UBC*, *UBA52*, and *RPS27A*,^94^ a process likely destined to support ubiquitin conjugation. It is worth noting that, since UBA52 and RPS27A are fused with ribosomal proteins, the processing of these gene products leads to the release of RPL40 and RPS27a. The up-regulation of these proteins may also serve as an indication of proteasome loss of function.

## Activation of the unfolded protein response and integrated stress response

Protein homeostasis is constantly monitored at the ER from which defective proteins are transported back to the cytosol for degradation. This process is named ER-associated degradation.^95^ Any decline in proteasome activity is accompanied by dysfunction of ER-associated degradation and concomitant accumulation of misfolded and/or damaged protein within the ER lumen. This agglomeration of protein aggregates in the ER activates the three ER membrane-resident receptors ATF6, PERK, and IRE1.^96^ These, in turn, trigger the so-called unfolded protein response, namely a compensatory transcriptional program which primarily aims at up-regulating molecular chaperones and ER-associated degradation components and whose cellular aspects have been already discussed elsewhere.^97^ Another key feature of the unfolded protein response is its ability to stop protein biosynthesis to prevent protein burden exacerbations. This translational arrest is mediated by PERK, which phosphorylates the initiation translation factor eIF2α. This post-translational modification inhibits GDP/GTP exchange by eiF2B.^98^ Importantly, eIF2α phosphorylation can also be catalyzed by three additional cytosolic serine–threonine kinases: general control non-derepressible-2, protein kinase R, and heme-regulated inhibitor. These kinases are part of the integrated stress response, a stress pathway activated in response to various pathogenic and/or proteotoxic stimuli.^99^ The relevance of the integrated stress response, protein kinase R in particular, as a reliable diagnostic biomarker for proteasome dysfunction has been recently highlighted in both CANDLE/PRAAS and neurodevelopmental proteasomopathies.^38^^,^^100^

## Type I IFN signatures

In this regard, protein kinase R is even more interesting, since besides phosphorylating eIF2α, it triggers a signaling cascade leading to the transcription of type I IFN genes.^101^ Accordingly, proteasome inhibition typically results in the up-regulation of type I IFN-stimulated genes such as *ISG15*, *IFI44L*, and *IFIT1 in vitro* in various cell types.^102^, ^103^, ^104^ Likewise, CANDLE/PRAAS and neurodevelopmental proteasomopathies have been consistently associated with persistent peripheral blood type I IFN gene signatures.^32^^,^^34^^,^^38^^,^^50^^,^^52^^,^^53^^,^^55^^,^^105^^,^^106^ Interestingly, Davidson et al recently highlighted the critical role of protein kinase R in initiating type I IFN response in these patients.^100^ In this study, the authors show that impaired protein breakdown is associated with cytosolic accumulation of IL-24, which is then sensed as a danger signal by protein kinase R. A notion that presupposes that autoinflammation triggered by proteasome dysfunction is limited to cells expressing IL-24. Although the activation of type I IFN responses aligns with CANDLE/PRAAS phenotypes, it does not correspond to the absence of typical clinical inflammation signs observed in patients with neurodevelopmental proteasomopathies. The discordance is not a completely novel paradox, as similar observations have been made in patients with Aicardi–Goutières syndrome and Down syndrome who exhibit increased IFN signaling despite lacking inflammatory symptoms.^107^

## Molecular pathogenesis of neurodevelopmental proteasomopathies

It has long been known that proteasome dysfunction affects both development and neuronal differentiation and/or function. This has been particularly well exemplified in animal models, notably mice in which knockout of the subunit genes *Psmd4*, *Psmd11*, *Psmg1*, *Psmc3*, and *Psmc4* is embryonic lethal (Table 3).^108^, ^109^, ^110^, ^111^ Inevitably, conditional knockout of the *Psmg1*, *Psmc1*, *Psmc4*, *Psmd4*, and *Psmd14* genes have been associated with severe neurological phenotypes characterized by brain atrophy, malformations, neurodegeneration, or neuron loss in various mouse and/or *Drosophila* models.^39^^,^^110^^,^^112^, ^113^, ^114^, ^115^, ^116^, ^117^ Supporting the importance of active proteasome function for CNS function, knockdown of the *Psmd11* and *Psmc5* genes resulted in growth retardation in mice and decreased learning ability in rats, respectively.^109^^,^^116^

**Table 3:** Mouse models for proteasome subunits.

Gene	Technique	Distribution	Phenotype	Reference
*Psmc1*	Tissue-specific conditional gene knockout (−/−)	Neurons	Neurodegeneration	117
	Conventional gene knockout (+/−)	Ubiquitous	Cell cycle defects	157
*Psmc3*	Conventional gene knockout (−/−)	Ubiquitous	Embryonic lethal	111
*Psmc4*	Conventional gene knockout (−/−)	Ubiquitous	Embryonic lethal	111
	Tissue-specific conditional gene knockout (−/−)	Muscles	Myofiber degeneration	158
	Tissue-specific conditional gene knockout (−/−)	Muscles	Muscle atrophy	159
*Psmc6*	Conventional gene knockout (−/−)	Ubiquitous	Protection against ovariectomy-induced osteoporosis	160
*Psmd4*	Conventional gene knockout (−/−)	Ubiquitous	Embryonic lethal	108
	Conditional PSMD4 ubiquitin-interacting motifs (UIM) knockout (−/−)	Ubiquitous	Embryonic lethal	
	Tissue-specific conditional PSMD4 ubiquitin-interacting motifs (UIM) knockout (−/−)	Liver	Disruption of protein homeostasis	
*Psmd5*	Conventional gene knock-in	Ubiquitous	Neurodegeneration	161
*Psmd11*	Conventional gene knockout (−/−)	Ubiquitous	Embryonic lethal	109
	Conventional gene knockout (+/−)	Ubiquitous	Growth retardation	
*Psmb8*	Conventional gene knockout (−/−)	Ubiquitous	Lipodystrophy	162
			Bipolar cell response defects	163
			Abnormal behavior	118
			MHC class I antigen presentation defects	164,165
			Neuroinflammation	166
*Psmb9*	Conventional gene knockout (−/−)	Ubiquitous	Neuroinflammation and neurobehavioral dysfunctions	119
			MHC class I antigen presentation defects	164,165
	Conventional knock-in of a Psmb9 variant (G156D/G156D)		Lethal	53
	Conventional knock-in of a Psmb9 variant (+/G156D)		Immunodeficiency	53
*Psmb11*	Conventional gene knockout (−/−)	Thymus	MHC class I antigen presentation defects	164,165
*Psmg1*	Tissue-specific conditional gene knockout (−/−)	Ubiquitous	Embryonic lethal	110
		Brain	Growth retardation and abnormal limb-clasping reflexes	
		Liver	Senescence	

Remarkably, while depletion of the immunoproteasome subunits *Psmb8* and/or *Psmb9* is primarily associated with MHC class I antigen presentation defects (Table 3), it fails to replicate the phenotype observed in CANDLE/PRAAS patients in terms of systemic autoinflammation. However, comprehensive investigations of the CNS in these mice have revealed a higher production of pro-inflammatory cytokines by astrocytes and/or microglia cells (Table 3), suggesting the occurrence of neuroinflammation. Most importantly, mice with a knockout of *Psmb8* or *Psmb9* exhibit some of the manifestations observed in patients with neurodevelopmental proteasomopathies, including dysfunctional behavior.^118^^,^^119^ These studies suggest that the overlap between autoinflammatory and neurodevelopmental proteasomopathies may be larger than initially assumed. Interestingly, as illustrated in Table 3, mice with a tissue-specific conditional knockout of the Psmc4 subunit exhibited muscle atrophy, thus linking 19S RP deficiency to reduced muscle tone.

Collectively, these mouse models underscore the significant role of proteasomes in both brain development and function. Nevertheless, our understanding of the pathogenesis of neurodevelopmental disorders remains rudimentary. Investigating the molecular mechanisms underlying these diseases is indeed hindered by the massive proteome changes occurring because of global impairment of protein breakdown. Taking this into account, the simplest hypothesis that could explain the neurological phenotype of individuals harboring proteasome loss-of-function variants is that such accumulation of protein aggregates — regardless of their origin — cannot be tolerated by the CNS. However, this would imply that the CNS is less efficient in coping with protein burden compared with other organs, an assumption for which there is little evidence thus far. Instead, it is tempting to speculate that defective proteasomes in these patients lead to the persistent expression and/or stabilization of critical regulators involved in developmental and neuronal pathways. As shown in Table 4, prominent proteasome substrates expressed in neurons include Hes1, Mov-10, Dab1, Cdk5 activator p35, Limk1, and Rap2a, all involved to varying degrees in the regulation of neurodevelopment. Of particular interest in this group is the negative regulator Limk1, whose persistent expression has been shown to limit axon elongation.^120^ Likewise, impaired removal of Cdk5 activator p35 by proteasomes leads to Cdk5 hyperactivation, a process that is associated with increased neurotoxicity in postmitotic neurons.^121^

**Table 4:** List of proteasome substrates involved in neurogenesis and/or neuronal function.

Function	Proteasome substrate	Ubiquitin ligase(s)	Reference
Neurodevelopment	Hes1	SCF^FBXL14^	167
	Mov-10	CRL4^DCAF12^	168
	Dab1	Cul5	169
	Cdk5 activator p35	Unknown	170,171
	LIM domain kinase 1	Rnf6	172
	Rap2A	Nedd4	173
Synaptic plasticity	Arc/Arg3.1	Ube3A, Triad3A	174,175
	Synaptophysin	Siah1	176
	Akap79/150	Unknown	177
Postsynaptic scaffolding	GKAP	Trim3	178
	Liprin-α	APC/C	179,180
	PSD-95	Mdm2	181
	Shank1	Unknown	177
	CNKSR2	Smurf2	182
	SPAR	SCF^β-TRCP^	183
	PTEN	Nedd4	184
	GRIP1	Unknown	185
Synaptic transmission	Syntaxin1	Rnf40	186
	GlyT2	LNX1 and LNX2	187
	mGluR1 and mGluR5	Siah1A	188
	GluK2	Cul3 actinfilin	189
	GluN1	Fbx2	190
	GluN2A	Mib2	191
	GluA1	Nedd4, Nedd4L, APC^Cdh1^	192, 193, 194, 195, 196
	GluA2	RNF167, RNF220	192,197,198
	GABA_A_Rβ3	Unknown	199
	nAChRα3	CHIP	200
	GlyR	Unknown	201
	DRD4	KLHL12	202
	DRD1, DRD5, and DRD2L	Unknown	202

Importantly, the breakdown of postsynaptic proteins by the UPS in response to neural activity is also a key process of both long-term potentiation and long-term depression, namely in learning and memory.^122^ For instance, degradation of Arc/Arg3.1 by proteasomes has been shown to preserve postsynaptic cell surface expression of AMPA-type receptors and thus maintain the strength of glutamatergic synapses resulting from long-term potentiation.^123^ Other proteasome substrates involved in this process include synaptophysin and Akap79/150, the latter accumulating in the brains of individuals with bipolar disorders.^124^ As shown in Table 4, the UPS also determines the molecular composition of the postsynaptic density,^125^ a massive and complex network made-up of membrane and cytosolic scaffolding proteins supporting synaptic interactions.^126^ Prime examples of postsynaptic density components targeted for proteasome-mediated degradation include PSD-95 and GKAP during dendritic spine remodeling. In addition, neurotransmitter receptors and/or transporters have their protein turnover regulated by the UPS (Table 4), thereby suggesting that proteasome dysfunction may profoundly alter synaptic transmission.

The formal determination of whether these proteasome substrates accumulate in the brains of patients with proteasome variants remains an area for future research. However, this question may be difficult to answer given that biological samples made available for research investigations usually consist of whole-blood specimens. Future studies will have to overcome this problem using neurons and/or brain organoids derived from induced pluripotent stem cells.

There has been a recent postulation that 19S RP exists as free and unbound complexes in both pre- and post-synaptic compartments in rats.^49^ Remarkably, this study proposes that 19S RP, when separated from their 20S CP, exhibit a UCHL-5-mediated de-ubiquitinating activity towards substrates modified with K63-linked ubiquitin chains. As a result, they regulate the trafficking of various synaptic proteins, including AMPA receptors, in a proteasome-independent manner.^49^ This observation represents a potentially ground-breaking development in our understanding of the molecular pathogenesis of neurodevelopmental proteasomopathies, assuming that 19S variants do alter the de-ubiquitinating activity of free 19S RP.

As discussed earlier, proteasome dysfunction is associated with the acquisition of specific molecular signatures (Fig. 3), and it is conceivable that persistent expression of either one these biomarkers may actively participate in disease etiology. For instance, chronic type I IFN responses have been associated with various detrimental effects on CNS function, including reduced serotonin production, impaired neurogenesis, increased demyelination, and neuronal cell death.^127^ Given the harmful impact of type I IFN on the CNS, it is reasonable to speculate that it plays a significant role as a disease driver in neurodevelopmental proteasomopathies, similar to its involvement in monogenic early-onset autoinflammatory encephalopathy Aicardi–Goutières syndrome, which is caused by mutations in the *TREX1*, *RNASEH2A*, *RNASEH2B*, or *RNASEH2C* genes.^128^ This assumption suggests that both Aicardi–Goutières syndrome and neurodevelopmental proteasomopathies might share the same underlying pathogenic determinants, although further research is required to confirm this hypothesis formally.

## Diagnosis of neurodevelopmental proteasomopathies

With the progressive implementation of whole-genome sequencing into routine clinical practice, the number of new cases of neurodevelopmental proteasomopathy is expected to increase in the coming years. The identification of new variants as well as the availability of larger patient cohorts will undoubtedly refine the genotype–phenotype correlation of these diseases. However, the greatest challenge in establishing proper diagnosis will remain the reclassification of variants of unknown significance (VUS), which typically requires additional functional tests.

To date, the only way to investigate the pathogenicity of VUS is to assess proteasome function in biological samples. Ideally, routine functional assays would allow reproducible and reliable quantification of proteasome activity as well as biomarkers associated with proteasome dysfunction, such as increased protein aggregation, mitophagy, and type I IFN responses in blood samples. The costly and time-consuming nature of such experiments revolving around methods such as native- and SDS-PAGE/Western-blotting make these explorations hardly suitable for rapid diagnostic purposes and for high-throughput classification of VUS.

The recent development of activity-based probes, small molecules that covalently bind to active sites of enzymes may offer an interesting alternative for testing VUS from proteasome genes. Using fluorescent activity-based probes, it is now indeed feasible to directly quantify enzyme activity through flow cytometry.^129^ Over the last couple of years, a large and constantly increasing number of fluorescent activity-based probes have been designed to monitor the activity of various proteases including proteasomes.^129^ Although the relevance of proteasome-specific activity-based probes for validating VUS remains to be fully explored, they seem suitable for one-day diagnosis of neurodevelopmental proteasomopathies from whole-blood samples. As discussed above, this strategy should be ideally accompanied by parallel quantification of relevant biomarkers of proteasome dysfunction using the same flow cytometer for a potential all-in-one platform. This objective can be achieved by using the fluorescent dyes PROTEOSTAT® and MtPhagy which measure protein aggregation and mitophagy respectively.^34^^,^^130^ In addition, the use of fluorescent monoclonal antibodies directed against cell surface proteins specifically up-regulated by type I IFN would allow a simultaneous evaluation of type I IFN signaling activity in the same samples by flow cytometry. These markers include notably SIGLEC1 (CD169), an adhesion molecule whose relevance for sensing interferonopathies has already been established.^131^ These experimental approaches should provide the complementary diagnostic procedure required for testing the functional significance of proteasome VUS newly identified by high throughput sequencing. Future studies in this respect will aim at standardizing techniques combining both genetic and functional testing for routine diagnosis of these rare diseases.

If biological samples are not readily available, another strategy involves testing whether the identified VUS can restore proteasome function in established cellular models that are deficient for the analyzed gene. Since all proteasome genes are essential, except for the immunoproteasome ones, this approach involves designing a drug-inducible gene knockout to deactivate the gene of interest at a specific point in time, shortly before the introduction of the VUS. While this approach would undoubtedly clarify the pathogenicity of a given VUS, it has significant limitations, including the necessity to pre-generate these models for each of the 52 proteasome genes and the possibility of overlooking tissue-specific effects.

One conceivable approach, powered by artificial intelligence, for analyzing proteasome VUS involves assessing their impact on the three-dimensional structure of the 26S proteasome. Currently, various tools, such as SIFT, PolyPhen-2, and MutationTaster, can help predict whether a variant is likely to be pathogenic or benign based on its location and effect on the individual protein. However, a notable limitation of these tools is their inability to consider the wider implications of a particular subunit variant within the entire 26S proteasome complex. Future research will need to explore the potential of utilizing cutting-edge, deep learning-based methodologies for protein complex prediction, such as AlphaFold-Multimer.^132^ These approaches hold indeed promise for conducting rapid *in silico* analyses, evaluating the pathogenicity of mutant subunits within the 26S proteasome complex.

## Therapeutics

Although cognitive impairment in neurodevelopmental proteasomopathies seems predetermined at birth and incurable, there is reasonable hope for the development of therapeutic strategies controlling disease progression and/or some of the symptoms in the near future. To date, the difficulty of designing such treatments lies in the lack of relevant molecular targets, a limitation due to our poor understanding of disease pathogenesis.

Even though the contribution of persistent type IFN responses to the acquisition of neurological disability remains to be fully determined, the development of autoinflammation — even subclinical — is one manageable symptom of these diseases. Targeting inflammation is a therapeutic approach used in other genetically determined interferonopathies of the CNS, including Aicardi–Goutières syndrome, which is caused by mutations in genes coding for proteins involved in nucleic acid processing and/or sensing.^133^ The treatment for Aicardi–Goutières syndrome involves the use of Janus kinase-specific inhibitors like baricitinib or ruxolitinib to hinder sustained type I IFN signaling.^134^, ^135^, ^136^ Although these therapies proved effective in alleviating skin manifestations,^137^ they were also associated with severe side effects, including leukopenia and recurrent infections.^135^ However, it remains unclear whether suppressing type I IFN would lead to significant improvement in cognitive function, and this observation raises the question of whether type I IFN is merely an epiphenomenon of the disease. Other therapeutics for Aicardi–Goutières syndrome include reverse transcriptase inhibitors and neutralizing antibodies.^133^ The full extent of the latter's potential relevance and applicability in the context of neurodevelopmental proteasomopathies is still awaiting comprehensive exploration.

In general, future treatment strategies should aim to target upstream type I IFN responses and seek to restore proteasome function. In this regard, one approach for restoring the activity of proteasomes with mutated subunits is to take advantage of the physiological processes up-regulating proteasome activity *in vivo*. However, our current knowledge about these mechanisms originates from a handful of studies only, a major drawback limiting the number of druggable pathways so far (Table 5).

**Table 5:** List of molecules described as proteasome stimulators.

	Molecule	Mode of action	Reference
Chemical compounds	IU1	USP14 inhibition	143
	Rolipram	Phosphodiesterase-4 inhibition	138
	Cilostazol	Phosphodiesterase-3 inhibition	139
	Sildenafil	Phosphodiesterase-5 inhibition	140
	Tadalafil		
	BAY41-2272	Guanylyl cyclase stimulation	
	Cinaciguat		
	PD169316	p38 MAPK inhibition	141
	SB202190		
	SB203580		
	PD169316		142
	AM-404	20S Gate opening stimulation	203
	MK-886		
	TCH-165		204
	Chlorpromazine	D2 dopamine receptor blockade	205
	Fluspirilene		206
	Dihydroquinazolines	Trypanothione reductase inhibition	207
	Nifenazone	Nonsteroidal anti-inflammatory drug	208
Natural substances	Betulinic acid	Unknown	209
	Curcumin	DYRK2 inhibition	145
	Oleuropein	Unknown	210
	Ursolic acid	p38 NF-κB inhibition	211
Recombinant proteins	Ubiquitinated MUC1	20S Gate opening stimulation	212
	Ubiquitin aldehyde		213
	Ubiquitinated E6AP		213
	ZFAND		214

For instance, cAMP-dependent protein kinase A and cGMP-dependent protein kinase G have been shown to accelerate the turnover of misfolded proteins by phosphorylating specific proteasome subunits.^138^, ^139^, ^140^ These observations provided a new basis for targeting enzymes regulating the intracellular pools of cAMP and cGMP second messengers in order to maximize proteasome function. As such, treatments with phosphodiesterase inhibitors or guanylyl cyclase stimulators have consistently been associated with increased proteasome function in various cell types, including neurons.^138^, ^139^, ^140^ As shown in Table 5, regulation of proteasome activity also occurs at the level of p38 MAPK, whose inhibition by various small-molecule inhibitors such as PD169316, SB202190, and SB203580 results in increased proteasome activity.^141^^,^^142^ Another regulator of proteasome function is the de-ubiquitinating enzyme USP14, whose inhibition by small-molecule inhibitor IU1 increases all three proteasome catalytic activities.^143^ Although the effects of IU1 exerted on peptide hydrolysis by 20S proteasomes seem convincing, its ability to accelerate the degradation of aberrant proteins found in neurodegenerative diseases remains controversial.^144^ Interestingly, some of the proteasome activators described so far also include chemical compounds used for the treatment of neurological and psychiatric disorders. These are notably chlorpromazine and fluspirilene (Table 5), which primarily antagonize D2 dopamine receptors and whose mode of action on proteasomes remains to be fully explored. Gate opening of the 20S complex may also represent a promising strategy to force protein breakdown in cells with defective proteasomes. As shown in Table 5, stimulators of 20S gate opening include the organic compounds AM-404, MK-886, and TCH-165, as well as recombinant proteins, among which ubiquitin-modified MUC1 and E6AP substrates. Besides organic compounds and recombinant proteins, a couple of natural substances have been shown to increase proteasome activity *in vitro*. As shown in Table 5, these include notably betulinic acid and curcumin, the latter impeding *PMSC3*/Rpt5 phosphorylation by targeting the DYRK2 kinase.^145^

Altogether, these studies offer an array of options for restoring proteasome function and combating protein aggregation in neurodevelopmental proteasomopathies. Future studies need to address the relevance of these strategies in cells directly isolated from patients, as well as in preclinical models.

## Conclusion

Neurodevelopmental proteasomopathies constitute an emerging group of Mendelian diseases, yet their complete clinical and molecular characterization remains an ongoing endeavor. Unfortunately, our understanding of their molecular pathogenesis is hampered by the implication of proteasomes in multiple — if not all — basic cellular processes. Although studies employing proteasome inhibitors have reliably described the primary cellular consequences of impaired protein breakdown, including proteotoxic stress, sterile inflammation, and remodeling of autophagy and lipid flux, these alone cannot account for the heterogeneity in phenotypes among neurodevelopmental proteasomopathies. Moreover, they offer limited explanations for the significant clinical variation observed within the broader category of proteasomopathies, which encompasses conditions like CANDLE/PRAAS. This leads to the hypothesis that variants in proteasome genes may exert effects extending far beyond their influence on proteasome catalytic activity. For instance, these additional effects could impact the stability of proteasomes, as well as their localization, post-translational modifications, or interactome. This point might be difficult to address considering the very low number of available biological samples worldwide and the rarity of the disease. In the meantime, research should take advantage of the increasing number of biomarkers associated with proteasome dysfunction for screening and therapeutic purposes.

## Author contributions

Conceptualization: S.C., S.B., S.K., and F.E.; data curation: S.C. W.D., V.V., and F.E.; writing—original draft preparation: S.C.; writing—review and editing: S.M., B.I. S.K., and F.E.; funding acquisition: S.B. and S.K. All authors read and agreed to the published version of the manuscript.

## Conflict of interests

The authors declare no conflict of interests.

## Funding

This work was supported by the European Joint Programme on Rare Diseases (EJP RD) for the project “UPS-NDDiag” and the Agence Nationale de la Recherche (ANR) for the project ANR-21-CE17-0005.
